# Waste Activated Sludge-High Rate (WASHR) Treatment Process: A Novel, Economically Viable, and Environmentally Sustainable Method to Co-Treat High-Strength Wastewaters at Municipal Wastewater Treatment Plants

**DOI:** 10.3390/bioengineering10091017

**Published:** 2023-08-29

**Authors:** Melody Blythe Johnson, Mehrab Mehrvar

**Affiliations:** Department of Chemical Engineering, Toronto Metropolitan University, 350 Victoria Street, Toronto, ON M5B 2K3, Canada; m5johnso@torontomu.ca

**Keywords:** WASHR, co-treatment, high-rate treatment, high-strength wastewater, waste activated sludge-high rate, winery wastewater

## Abstract

High-strength wastewaters from a variety of sources, including the food industry, domestic septage, and landfill leachate, are often hauled to municipal wastewater treatment plants (WWTPs) for co-treatment. Due to their high organic loadings, these wastewaters can cause process upsets in both a WWTP’s liquid and solids treatment trains and consume organic treatment capacity, leaving less capacity available to service customers in the catchment area. A novel pre-treatment method, the Waste Activated Sludge-High Rate (WASHR) process, is proposed to optimize the co-treatment of high-strength wastewaters. The WASHR process combines the contact stabilization and sequencing batch reactor processes. It utilizes waste activated sludge from a municipal WWTP as its biomass source, allowing for a rapid start-up. Bench-scale treatment trials of winery wastewater confirm the WASHR process can reduce loadings on the downstream WWTP’s liquid and solids treatment trains. A case study approach is used to confirm the economic viability and environmental sustainability of the WASHR process compared to direct co-treatment, using life-cycle cost analyses and greenhouse gas emissions estimates.

## 1. Introduction

Municipal wastewater treatment plants (WWTPs) are often tasked with treating loads of high-strength wastewaters, such as septage, landfill leachate, and industrial wastewaters [1,2]. Commonly, these high-strength wastewaters are hauled to municipal WWTPs by truck, allowing operators to control their addition rates and locations for the overall treatment process.

Full-scale anaerobic co-digestion with municipal wastewater sludges was shown to be effective for the treatment of a wide variety of wastewaters from the food industry, including fats, oils, and greases (FOG); dairy manufacturing wastewater; slaughterhouse wastewater; and winery wastewater (WWW), among others [3,4,5,6,7,8]. Wastewaters generated by the food industry are often also biodegradable by aerobic biomass, and co-treatment with domestic wastewater in the aerobic bioreactors at municipal WWTPs was also successfully used for co-treatment [9,10]. Septage and leachate are often co-treated in a municipal WWTP’s liquid treatment train [2].

Despite this, there are several factors that limit the feasibility of the direct co-treatment of high-strength wastewaters using the existing municipal WWTP infrastructure. Even small volumes of high-strength wastewater can exert a substantial organic loading in a WWTP’s treatment trains, reducing the capacity available for domestic wastewater servicing. In addition, without adequate equalization infrastructure, the rapid off-loading of hauled waste truck contents into the treatment train can cause slug loadings, which can negatively impact both the aerobic and anaerobic treatment processes. Finally, significant variations in influent loadings can negatively impact process performance, with potential impacts including anaerobic digester souring due to organic overloading; poor effluent quality due to insufficient aerobic reaction time and/or exceeding the available oxygenation system’s capacity; high sludge-generation rates impacting the operating solids retention time (SRT) and nitrification performance; and poor sludge settleability impairing the secondary clarifier solids capture and the biomass inventory capacity of the aerobic bioreactors. These negative impacts were observed during the co-treatment of WWW [5,11,12], a high-strength wastewater that can exert significant environmental impacts on receiving water bodies even after treatment [13,14].

The Niagara Region (Region) is the hub of the wine industry of Ontario, Canada, which is home to over 100 wineries [15] and provides over 90% of Canada’s grape-growing capacity [16]. During the vinification season, which coincides with the start of the grape-harvest period in the late summer, the Region’s municipal WWTPs are subject to significant increases in the quantity and strength of the WWW hauled in for co-treatment [17]. The current operating strategy of the direct co-treatment of the WWW in either the anaerobic digesters or liquid treatment train results in negative operational and performance impacts [18]. A more effective approach to WWW handling and treatment at the Region’s WWTPs is required to ensure continued compliance with WWTPs’ effluent standards as the wine industry and serviced populations continue to grow.

The overall objective of this study was to develop a pre-treatment system for hauled high-strength wastewaters capable of reducing loadings on downstream municipal WWTPs’ treatment trains that is simple to operate, minimizes or eliminates the need for additional chemicals, and is economically viable and environmentally sustainable. To be able to react to the rapidly changing influent loading conditions associated with the batch discharge of hauled wastewaters, the process must be capable of a rapid start-up and produce an effluent that is suitable for subsequent treatment in the municipal WWTPs’ solids and/or liquid treatment trains.

A novel pre-treatment method, the Waste Activated Sludge-High Rate (WASHR) process, was developed to meet these objectives, and an international patent application was filed for this novel technique [18]. The process utilizes two waste streams from the municipal WWTP process and a batchwise treatment in stand-alone tankage to reduce organic loadings into downstream WWTPs’ treatment trains. The performance of the WASHR process was confirmed at the bench scale for the treatment of high-strength WWW, and an economic analysis was completed using a case study to confirm its cost effectiveness.

## 2. Methodology

### 2.1. Analytical Methods

In situ reactor dissolved oxygen (DO), temperature, and pH were measured using LDO101 and PHC101 probes connected to a portable HQ30d meter (HACH, London, ON, Canada). Total solids (TS), volatile solids (VS), total suspended solids (TSS), and volatile suspended solids (VSS) were analyzed per APHA Section 5210B and Section 2540, as appropriate [19]. Samples were filtered using 0.45-micron glass Whatman filter papers.

HACH method 8000 was used for chemical oxygen demand (COD). A Shimadzu TOC-500A (Shimadzu, Columbia, MD, United States) analyzer was used for total organic carbon (TOC). A Skalar SAN Plus 3000/5000 Segmented Flow Analyzer (Skalar Analytical B.V., Breda, Netherlands) was used for total ammonia nitrogen (TAN) (method 155–324 w/r), total phosphorus (TP), and ortho-phosphate phosphorus (PO_4_-P) (method 503–324 w/r).

### 2.2. Winery Wastewater and Anaerobic Digester Supernatant

A sample of actual WWW was directly collected from a load hauled by truck to a municipal WWTP in Niagara Region on 7 October 2019. Digester supernatant was directly collected from a secondary (unheated and unmixed) anaerobic digester from a Niagara Region municipal WWTP on 8 October 2019. Both the WWW and digester supernatant were analyzed per the methods outlined in Section 2.1, and their characteristics are presented in Table 1.

### 2.3. Mixed Liquor

Mixed liquor was collected from the aerobic bioreactors of one of Niagara Region’s conventional activated sludge (CAS) municipal WWTP on 9 October 2019. The facility has a rated capacity of 61,350 m^3^/d and is equipped with primary sedimentation tanks located upstream of the aerobic bioreactors, with oxygenation provided by mechanical aeration. Samples of mixed liquor were directly collected from the bioreactors on the morning of the bench-scale trials.

### 2.4. Experimental Set-Up and Procedures

Bench-scale trials were conducted using 20-L flat-bottomed, open-topped containers with a diameter of 29 cm, each equipped with two ceramic fine bubble diffusers, one 9 cm in diameter and the other 4 cm in diameter. A total aeration rate of 4.0 L/min per container was provided by an 8-outlet ActiveAQUA AAPA25L air pump (Hydrofarm, Petaluma, CA, USA). Reactors were operated at room temperature, with recorded temperatures varying from 16.3 to 17.4 °C during testing of the contact stage and from 11.1 to 16.7 °C during the stabilization stage. Each aerobic reactor was seeded with 14 L of waste activated sludge (WAS) directly taken from a municipal WWTP’s bioreactor. The reactors were fed with actual WWW and anaerobic digester supernatant during the contact stage of the process, with WWW added at time zero, and supernatant added at 2 h. Sodium hydroxide diluted with distilled water to 1 N was used to adjust the pH of the WWW to 8 prior to its addition to the reactors.

Over the course of the contact stage test, 1 L of supernatant and 1.7 L of mixed liquor were removed from each reactor for analysis. Prior to the stabilization stage, supernatant was removed until only 6 L of settled biomass remained in each bioreactor.

Four bench-scale reactors were run in parallel at increasing loadings of WWW (Runs A, B, C, and D operating at WWW loadings of 1%, 2%, 3%, and 4% *v*/*v*, respectively). Anaerobic digester supernatant was also added to each run (3.9% *v*/*v*) as an additional nutrient (N and P) source, while the pH of the WWW was adjusted to 8.0 using 1 N NaOH prior to its addition to each reactor. Table 2 presents detailed operating conditions for the bench-scale trials.

## 3. Configuration and Performance of the WASHR Process

### 3.1. WASHR System Configuration, Theory, and Concept

**Figure 1:**
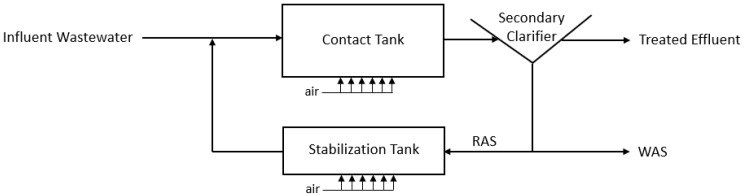
Process flow diagram of the contact stabilization activated sludge process.

The WASHR process is based on the contact stabilization (CS) activated sludge process that was a popular treatment scheme for municipal WWTPs in the mid-20th century but was phased-out in most jurisdictions as effluent quality requirements became more stringent [2]. In its full-scale configuration, CS consisted of a contact tank where influent wastewater was allowed to mix with activated sludge. The residence time in the contact tank was short, generally ranging between 0.5 h and 3 h, providing enough time for contaminant sorption with the biological floc [2,20]. The solids separation of the activated sludge from the secondary effluent was accomplished in a clarifier, with treated effluent directed to additional treatment steps. To maintain consistent mixed liquor concentrations, WAS was periodically wasted from the system as needed. The return activated sludge (RAS) was pumped into an aerated stabilization tank, which provided the oxygenation and reaction time necessary for the oxidation of the organic material sorbed to the activated sludge floc. The removal efficiencies for 5-day biochemical oxygen demand (BOD_5_) were generally in the range of 85% to 95% for domestic wastewater; however, due to the high-rate nature of the CS system, this process provided little to no nitrification [2,20]. Figure 1 presents a process diagram of the conventional high-rate contact stabilization process.

The WASHR process utilizes the contact stabilization concept modified in several novel ways to operate as a dedicated pre-treatment system for high-strength wastewaters. Rather than seeding and maintaining a dedicated biomass within the WASHR process, each treatment cycle utilizes newly wasted WAS from a municipal WWTP’s liquid treatment train. This allows the system to be brought online when and as needed, with no start-up or acclimation period required. Due to the nature of the CS process, the WASHR process can operate at a high food-to-microorganism ratio, minimizing the required operating mixed liquor suspended solids (MLSS) concentration. However, the overall treatment capacity of the WASHR process is limited by the available daily WAS wasting rate from the WWTP’s main liquid treatment train.

Nutrient limitations that can affect the performance of the aerobic biological treatment are common in high-strength wastes, such as insufficient N and P in WWW [2]. To treat high-strength wastewaters that are deficient in N and/or P, the ammonia- and phosphorus-rich supernatant from the municipal WWTP’s anaerobic digestion process can be added to the WASHR process as a nutrient source, without the need for additional chemicals. If anaerobic digester supernatant is not available, a chemical addition could be used to add additional nutrients as needed.

In addition, while the CS process operates in a continuous mode, the WASHR process operates in a batch mode to better accommodate the periodic loads of the high-strength wastewaters hauled to the WWTP by tanker trucks. Batchwise operation also allows for operational flexibility, to vary the duration of the aerobic biological treatment during both the contact and stabilization stages. The WASHR contact tank operates in a five-step mode similar to that of a sequencing batch reactor (SBR):

- Step 1: fill cycle: WAS and high-strength wastewater are added to the contact tank; if necessary, the municipal WWTP’s effluent can be added to dilute the WAS.
- Step 2: react cycle: the contact tank is continuously aerated, during which time organic and other contaminants are sorbed onto the biological floc.
- Step 3: settle cycle: aeration ceases, and the contact tank contents are allowed to settle under quiescent conditions.
- Step 4: empty cycle: the clarified supernatant is directed to the liquid treatment train of the municipal WWTP, and the settled biomass is transferred to the WASHR stabilization tank; at the end of this four-step process, the WASHR contact tank is empty and ready for another batch treatment cycle.
- Step 5: idle cycle: the empty contact tank awaits the start of the next fill cycle.

The WASHR stabilization tank provides aerobic oxidation of the organic material captured by the biological floc during the contact stage, in a four-stage process:

- Step 1: fill cycle: during the empty cycle of the contact stage, settled biomass is directed to the stabilization tank, which marks the start of the stabilization stage’s fill cycle.
- Step 2: aeration cycle: the settled biomass is continuously aerated, allowing the biomass to continue oxidizing the organic material sorbed during the contact phase.
- Step 3: empty cycle: the settled, stabilized biomass is emptied out of the stabilization tank and directed to the municipal WWTP’s digestion process for further treatment.
- Step 4: idle cycle: the empty stabilization tank enters the idle cycle, awaiting the transfer of the settled biomass from the empty cycle of the next contact stage.

**Figure 2:**
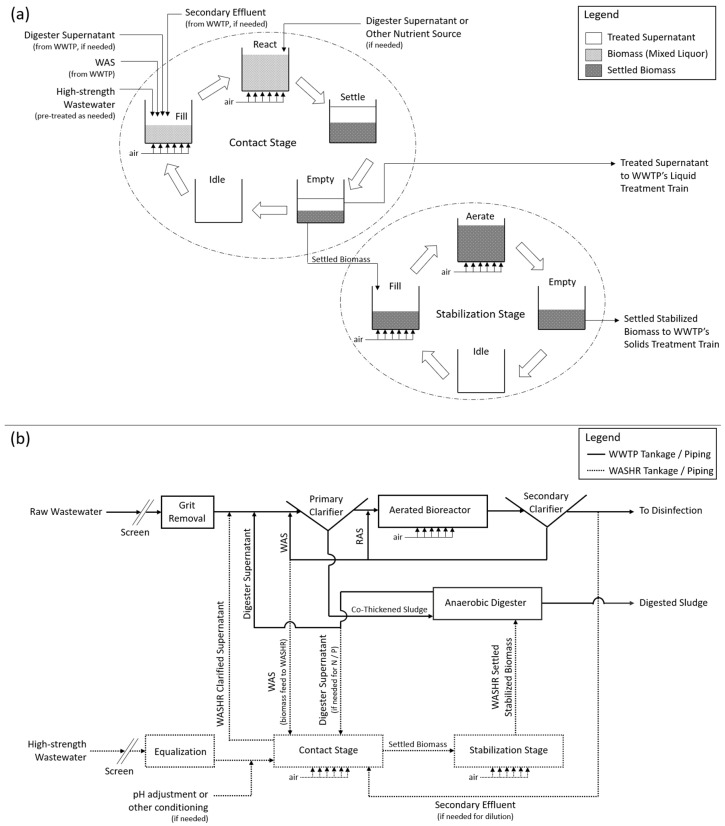
WASHR process: (**a**) detailed process flow diagram and (**b**) simplified process flow diagram showing its integration into an existing CAS municipal WWTP treatment process.

Figure 2a presents a process flow diagram of the WASHR system, while Figure 2b presents a simplified process flow diagram of the WASHR system integrated into a CAS municipal WWTP treatment process that co-thickens WAS. The WASHR process could also be accommodated within any suspended growth system that generates a WAS stream, including but not limited to extended aeration, a membrane bioreactor (MBR), and integrated fixed-film activated sludge processes.

The WASHR process is best-suited for high-strength wastewaters that have components that are rapidly removed via sorption onto the biological floc and have a significant readily biodegradable fraction (either aerobic or anaerobic). While a portion of the aerobically biodegradable fraction is removed during the WASHR process, the anaerobically biodegradable fraction is removed during the anaerobic digestion of the WASHR waste sludge in the WWTP’s anaerobic digesters.

**Figure 3:**
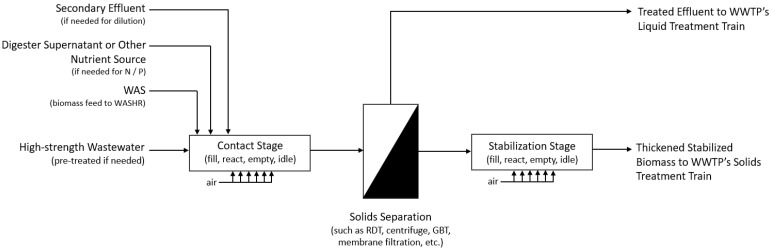
Potential modification to the WASHR process treating wastewaters that negatively affect sludge settleability.

In addition, the WASHR process could operate with minimal equipment and operational complexity for wastewaters that do not negatively affect the settleability of the activated sludge, allowing a simple, single-tank configuration for the contact stage of the process. For wastewaters that negatively impact settleability, the settle cycle of the contact stage could be replaced with a dedicated solids separation process. A mechanical sludge thickening process, such as a rotary drum thickener (RDT), thickening centrifuge, or gravity belt thickener (GBT), could be installed to provide solids separation at the end of the contact phase. An MBR could also be used for the contact stage, providing simultaneous solids separation and sludge thickening within the contact tank. These potential process modifications are presented in Figure 3 and demonstrate the flexibility of the WASHR process.

### 3.2. Bench-Scale Trials

#### 3.2.1. Contact Stage Performance

**Figure 4:**
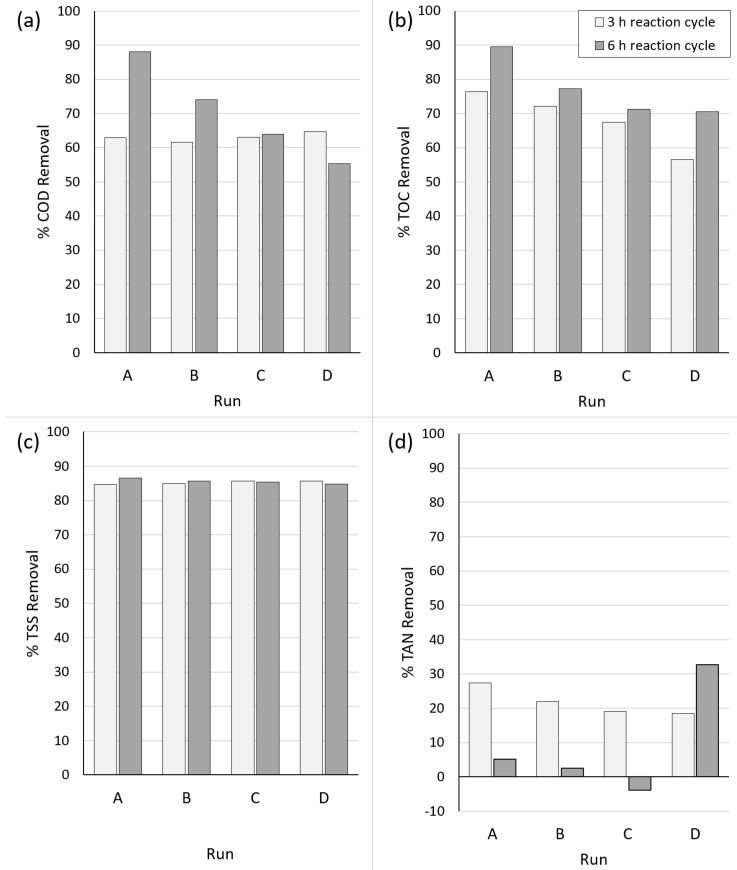
Results of removal efficiencies during the contact phase of the bench-scale WASHR treatment of WWW after reaction cycle times of 3 h and 6 h for (**a**) COD, (**b**) TOC, (**c**) TSS, and (**d**) TAN.

Figure 4 presents the removal efficiencies of COD, TOC, TSS, and TAN during the contact treatment phase of the bench-scale WASHR process for all treatment runs. The removal rates were based on the initial parameter concentrations shown in Table 2 and the measured clarified effluent concentrations after 3 h and 6 h of reaction time.

After 3 h of reaction time, the COD removal rates were within a narrow range, from 61.5% to 64.6%, for all runs, regardless of COD_o_, which ranged from 1751 to 6639 mg/L (Figure 4a). After 6 h of reaction time, the COD removal rates further increased for the reactors subjected to the lowest initial loadings (Runs A and B) but decreased for the reactor with the highest loading (Run C). Conversely, the 3-h TOC removal rates decreased as TOC_o_ increased, varying from 56.6% (TOC_o_ 2302 mg/L) to 76.3% (TOC_o_ 583 mg/L), with further improvements in TOC removal after 6 h of reaction time for all runs (Figure 4b). These results suggest the rapid sorption of the WWW constituents that contribute to COD and TOC, which is consistent with the finding of previous bench-scale trials using mixed liquor from four municipal activated sludge WWTPs. However, the COD and TOC removal rates cannot be simultaneously maximized.

The TSS removal rates (Figure 4c) were consistent for all runs, regardless of the reaction time, with removal rates ranging from 84.7% (Run A at 3 h) to 86.5% (Run A at 6 h). A rapid (<6 h) incorporation of WWW solids into the biological floc was observed during previous co-treatment trials; the results of the WASHR trials suggested that WWW solids are incorporated within 3 h and that the mixed liquor can accommodate initial TSS loadings of up to 2.3 mg TSS_o_/mg MLVSS without a deterioration in TSS removal.

The TAN removal rates at 3 h (Figure 4d) varied from 18.5% (Run D) to 27.3% (Run A). After 6 h of contact time, the removal rates significantly decreased for Run A and Run B, decreasing to 5.1% and 2.1%, respectively. Furthermore, the calculated removal rate for Run C at 6 h was negative (−3.8%), with the clarified supernatant TAN concentration (33.0 mg/L) higher than TAN_o_ (31.8 mg/L). Conversely, the TAN removals for Run D increased from 18.5% at 3 h to 32.7% at 6 h. The MLSS used for the WASHR trials was from a non-nitrifying municipal WWTP. The data suggest that both the biological uptake of TAN for biomass growth and the conversion of organic nitrogen to TAN are occurring during the contact phase. At lower COD_o_:MLVSS ratios (<4.0 mg COD/mg MLVSS), the biological uptake of TAN appears to predominate at a contact time of up to 3 h; however, by 6 h, the conversion of organic nitrogen to TAN appears to predominate. At the highest tested COD_o_:MLVSS ratio (5.3 mg COD/mg MLVSS), biological uptake appears to predominate throughout the entire 6 h contact time.

The TP removal rates were also evaluated. For all runs, the TP removals were negative at both 3 h (ranging from −26% to −103%) and 6 h (ranging from −16% to −116%). The reason for the apparent negative TP removal is unclear; however, as with organic N, it is possible that particulate P was being converted to soluble species during the contact stage. Despite this, the TP concentration appeared to exceed that which was initially fed to the reactors (Table 2). This may be due to the presence and/or release of phosphorus present in the mixed liquor, which was not accounted for in the initial feed concentrations. The concentration of PO_4_-P, which is highly bioavailable, ranged from 0.16 mg/L (Run A) to 0.42 mg/L (Run D) after 6 h, suggesting that solubilization may be contributing to the increase in supernatant P concentration and confirming that P was not a limiting nutrient in the trials.

#### 3.2.2. Stabilization Stage Performance

During the stabilization stage, the settled biomass from the contact stage is aerated (Figure 2a) to provide additional pre-treatment prior to the discharge of these solids into the municipal WWTP’s solids treatment train (Figure 2b). During the contact stage, COD is removed via sorption, TSS removal is enhanced by the incorporation of solids into the biological floc, and some biological oxidation is provided. The performance assessment of the WASHR process stabilization stage focuses on the TS, VS, TSS, and COD removal rates, since these are key parameters that affect the design requirements and performance of the WWTP’s digestion process, which is the ultimate end point for the settled biosolids after stabilization. The characteristics of the settled biomass both pre- and post-stabilization for the key parameters are shown in Table 3.

TS removal was observed for all runs, ranging from 7.4% (Run A) to 16.6% (Run D). The VS removal rates were lower, reaching a maximum of 11.6% for Run C, with a small increase in VS observed for Run A. Conversely, all runs showed negative TSS removal rates, with TSS concentrations increasing from 9.3% (Run A) to 13.1% (Run B). It can be inferred from these results that there is active biomass growth during the stabilization stage resulting in an increase in TSS, with a simultaneous reduction in VS and TS due to the conversion of a portion of these solid fractions to inert, volatile, or gaseous products during aerobic biological oxidation.

Negative COD removal was observed for Run A (−21.7%), and there was essentially no removal (0.8%) for Run B. This may be due to the conversion of complex compounds that resist oxidation during the COD test into more readily oxidizable products, resulting in an apparent increase in COD. Therefore, the observed COD removal rates should be evaluated in terms of any trends that can be observed, rather than in terms of the absolute values. The COD removal rates increased for runs with higher initial COD loading rates in the contact phase, appearing to plateau for Run C and Run D (contact phase loadings of 4.0–5.3 mg COD/mg MLVSS).

### 3.3. Discussion and Analysis

The objective of the WASHR process is to provide the pre-treatment of high-strength wastewaters to reduce loadings on the municipal WWTP. Typical co-treatment involves the direct discharge of high-strength wastewaters into either the solids or liquid treatment train. Conversely, the liquid-phase WASHR effluent is treated in the bioreactor, and the solid-phase WASHR effluent is treated in the solids treatment train (Figure 2b).

Actual WWW was used as a complex and high-strength wastewater stream to assess the effectiveness of the WASHR process at the bench scale. Given WWW’s low biochemical oxygen demand (BOD)-to-COD ratios, a significant fraction of the COD may be resistant to aerobic treatment [5,21,22]. Full-scale anaerobic treatment readily removes a significant fraction (89%) of the initial COD [5]. In addition, our own study of kinetics and co-treatment, completed in separate experiments, showed that sorption is a key contaminant removal mechanism during the aerobic biological oxidation of WWW by activated sludge from municipal WWTPs, with COD removal rates of up to 98% at initial concentrations of up to 1550 mg/L. Furthermore, our earlier bench-scale trials confirmed that pH-inhibition effects could be eliminated by adjusting the WWW’s pH prior to addition to the aerobic biomass [23]. The results of the bench-scale trials of the WASHR process confirmed that high removal rates can be achieved using aerobic biomass and relatively short (3–6 h) contact times. In addition, despite the low BOD:COD ratio of WWW, the high removal rates of both BOD and COD can be concurrently achieved using the aerobic biomass in the WASHR process.

The results of the treatment trials suggested that initial COD loadings did not impact COD removal rates at 3 h of contact time; however, at 6 h, the reactors operated at higher initial loadings (Run C and Run D at 4.0 mg and 5.3 COD_o_/mg MLVSS, respectively) and showed no improvement or a reduction in COD removal. Conversely, TOC removal was a function of both the initial loadings (mg TOC/mg MLVSS) and contact time. Over the range of the tested initial TOC loadings (0.47 to 1.9 mg TOC_o_/mg MLVSS), the TOC sorption increased with increasing reaction time and a lower initial TOC-to-biomass loading. Therefore, it is not possible to select operating conditions that can simultaneously optimize the performance of the contact stage in terms of COD removal, TOC removal, and minimizing the reactor volume (by increasing the applied loading rates).

A holistic assessment approach was used to calculate the reductions in loadings into the municipal WWTP’s liquid and solids treatment trains, which could be achieved via the use of the WASHR pre-treatment system compared to directing all the WWW to either treatment train (the current operating strategy). The parameters of focus of each treatment train consist of their critical design parameters. The loading reductions estimated using the results of the bench-scale trials are presented in Table 4. These results are based on reaction times of 3 h (contact phase) and 21 h (stabilization stage).

Pre-treating WWW using the WASHR system, rather than discharging this high-strength wastewater directly into the WWTP’s liquid treatment train, has the potential to reduce COD and TOC loadings by more than 81% and TSS loadings by more than 92%, thus minimizing the potential negative impact of co-treatment on the liquid treatment train. For example, if WWW is typically added upstream of the primary clarifiers, this would reduce the potential for septic conditions that can cause odors and rising sludge in the clarifier. No matter where the point of addition to the liquid treatment train is, WWW has a significant impact on the operation and performance of the downstream aerobic bioreactors due to its high soluble COD concentration and minimal removal across primary clarification. The operational issues encountered include a low operating DO concentration and increases in solids yield, affecting the operating solids retention time (SRT).

Pre-treating WWW using the WASHR system, rather than discharging this high-strength wastewater directly into the WWTP’s solids treatment train (digestion processes), has the potential to reduce COD loadings by more than 59% and VS loadings by more than 47%. These reductions in loadings reduce the potential for digester souring, which is a risk when co-digesting high-strength wastewaters such as WWW [5], particularly when the loadings from these high-strength wastewaters have significant temporal variations [1,2,3].

Based on the results of the bench-scale trials, the WASHR process can significantly reduce the loadings on downstream municipal WWTP treatment processes. The operating costs are greatly reduced because this process uses two waste streams (WAS and digester supernatant) to provide biomass and nutrients to the system. In addition, the substantial reductions in loadings into the municipal WWTP’s liquid and solids treatment trains can reduce and/or eliminate the upgrades needed to effectively treat high-strength waste streams, particularly those that are periodic in nature. While the treatment trials of the WASHR process have thus far focused on WWW, they should be expanded to other wastes that are often hauled to municipal WWTPs, including septage, portable toilet waste, feed mill wastewater, dairy wastewater, and slaughterhouse wastewater, among others.

Despite the above, the WASHR system has several limitations. Its overall daily treatment capacity is limited by the daily mass of the WAS generated by the municipal WWTP’s liquid treatment train. Furthermore, for facilities that do not have a nutrient-rich internal recycle stream (such as anaerobic digester supernatant), additional chemicals may be required which would increase the operational complexity and cost of the WASHR system.

## 4. Economic and Environmental Impact Analysis

### 4.1. Capital and Operating Costs

The development of the capital cost estimates associated with the implementation of the WASHR process are site-specific and depend on the nature and volume of the high-strength wastewater to be treated, as well as the configuration of the municipal WWTP and the characteristics of its mixed liquor. In addition, although direct co-treatment in the WWTP’s solids and/or liquid treatment trains is often accomplished without implementing any upgrades, this is accomplished at the cost of the available treatment capacity [5]. To maintain the available capacity to accommodate influent flows and loadings from a WWTP while also co-treating hauled high-strength wastewater, it is necessary to expand the capacities of the key unit processes. These generally include expanding the aerobic bioreactor and anaerobic digester volumes as well as the amount of ancillary equipment (blowers for bioreactors; heat exchangers, mixing systems, and biogas handling systems for digesters). Developing these upgrade needs also needs to be assessed on a case-by-case basis, taking into consideration the current and projected future influent flows and loadings requiring treatment, the effluent targets that need to be maintained, and the minimum design requirements as specified by local regulatory agencies.

Differential operations and maintenance (O&M) costs are driven by the energy needs to aerate the aerobic bioreactors of the WASHR process and/or the WWTP’s liquid treatment train, as well as the heating and mixing of the anaerobic digesters. The biogas generated during the co-digestion of high-strength wastewaters can be utilized for heating, which could offset some or all the additional anaerobic digester’s heating costs. In the case of treating high-strength wastewaters that are seasonal in nature, such as WWW, the digesters are oversized during low-loading periods; however, they still require heating and mixing, increasing year-round O&M costs. As with capital costs, O&M cost estimates depend upon the unique characteristics of the WWTP facility and the high-strength wastewater to be treated.

The advantage of the WASHR process is that it can accommodate seasonal variations in loadings, if needed, since its start-up is immediate, and all necessary inputs are available. This is particularly important for wastewaters with seasonal variations in loadings, such as WWW. At the same time, properly co-treating WWW directly in either the liquid or solids treatment train not only requires providing additional aerobic bioreactor and/or anaerobic digester volume, it also necessitates building up sufficient biomass in advance of the high-loading season to accommodate the increased number of loadings. Therefore, adding a food source to ensure biomass availability prior to the vintage season is used for systems treating variable WWW loadings [24], which needs to be considered in the O&M cost estimates for any co-treatment scheme.

For the purpose of this economic analysis, the capital cost estimates were developed using typical engineering design approaches, including unit costs for excavation, fill, and cast-in-place concrete, estimates for major process equipment including installation and engineering, with contingency allowances at 30%. The operations and maintenance (O&M) costs were estimated using the typical costs for electricity (CAD 0.14/kWh, per the Niagara Region), and the supplemental feed to the digester and/or bioreactors to increase the biomass prior to the vintage season was assumed to be sugar with a bulk cost of CAD 578/ton [25]. The life-cycle cost (LCC) estimates were based on a 25-year period, discount rate of 4%, and energy cost escalation rate of 2% per annum. All costs are reported in 2021 and are in Canadian dollars. A case study is presented in a later section.

### 4.2. Quantifying Greenhouse Gas Emissions

Greenhouse gas (GHG) emissions quantification needs to consider the configuration and operation of the WWTP, the methods used for biological oxidation (aerobic and anaerobic), the overall loadings of high-strength wastewater, and the efficiencies and emission rates of the biological and other processes used. For the WASHR process, the GHG emissions from not only the pre-treatment system but also the impact of the treated effluent (sent to the liquid treatment train) and waste biomass (sent to the anaerobic process) must also be considered for a holistic assessment of the overall impacts on GHG emissions. Similarly, the direct co-treatment of WWW in the municipal WWTP must take into consideration the impact on the aerobic bioreactors and anaerobic digesters.

Operating large equipment, such as blowers, requires considerable energy use in the form of electricity. The amount of consumed electricity can be converted into a measure of equivalent CO_2_ (eCO_2_), which varies geographically based on the type of power source available (e.g., higher eCO_2_/kWh for coal-fired plants vs. hydroelectric plants). For Ontario, this value is 0.029 kg eCO_2_/kWh [26].

GHG emissions are also produced during biological treatment: CO_2_ and N_2_O are released during aerobic treatment, while CO_2_ and CH_4_ are released during anaerobic treatment. The mass of each GHG emitted depends on the operation of the system as well as the influent loadings. For aerobic treatment, the mass of the CO_2_ emitted by heterotrophs per kg of BOD removed was reported to be 0.14 kg CO_2_/kg of BOD [27]. N_2_O, a much stronger GHG than CO_2_, is generated during nitrification in the aerobic activated sludge process but is not released at appreciable levels by facilities that are not nitrifying, thus contributing little to the overall GHG emissions [27,28,29]. Anaerobic digesters generate a great deal of biogas (CH_4_), which is typically used as a fuel source for the boilers maintaining adequate operating temperatures in the digesters, with the excess biogas flared to convert CH_4_ to CO_2_, reducing the GHG potential as well as increasing safety. Current biogas flares can vary in terms of performance with respect to local conditions such as the methane content of the gas being flared, the configuration of the flare stack, and the local wind conditions [30,31]. Generally, well-operated flares can be assumed to have conversion efficiencies of 96%–98% [30]. A value of 98% was used for this assessment.

### 4.3. Case Study

The feasibility of the full-scale implementation of the WASHR process was confirmed through a case study analysis. The upgrade needs were developed to provide treatment of an average of 40 m^3^/d (peak of 60 m^3^/d) of WWW during the vintage season, for a total seasonal volume of 4800 m^3^ or approximately 30% of all the WWW generated in the Region of Niagara. Based on the average characteristics from the WWW samples collected in the Region [17], this would result in estimated average COD, TSS, and VSS loadings during the vintage season of 3400 kg/d, 496 kg/d, and 1228 kg/d, respectively. These loading rates were used as the design basis for the economic assessment of all the treatment methods. Furthermore, it was assumed that these loadings would occur over a 4-month vintage period.

The case study facility was a conventional activated sludge WWWP with anaerobic digestion located in the Region. The facility has a rated capacity of 61,350 m^3^/d and is currently operating at approximately 57% of its rated capacity. The WWTP generates, on average, approximately 2000 kg/d of WAS with an average MLVSS:MLSS ratio of 0.76.

The upgrades required for the WWTP’s liquid and solids treatment trains were developed for three treatment options (pre-treatment using the WASHR process; direct co-treatment in the liquid treatment train; and direct co-treatment in the solids treatment train) for each timeline scenario. These upgrade needs were determined based on the projected influent loadings and maintaining operating conditions within the standard design guideline values [1]. An allowance for the construction and operation of a new hauled waste station, including coarse screening, grit removal, equalization (30 m^3^), and pH adjustment, was incorporated into all options. The bioreactor and/or anaerobic digestion capacity increases were assessed for each option, and the capital cost estimates included allowances for increasing the capacity of associated equipment, such as boilers, gas flares, digester mixing equipment, blowers, diffuser assemblies, piping, instrumentation, and other appurtenances.

In addition to the treatment options, two future loading scenarios were also considered: short-term, representing the upgrades needed to address co-treatment limitations assuming modest growth (15%) in the overall influent flow rates to the WWTP; long-term, representing the upgrades needed when the WWTP is operating at 100% of its rated capacity.

The conceptual design of the WASHR process was based on a contact phase COD loading rate of 4.0 kg COD/kg MLVSS, an operating MLSS concentration of 2000 mg/L, an MLVSS:MLSS ratio of 0.76, a total contact phase duration of 5 h (0.5 h fill, 3 h react, 0.5 h settle, and 1.0 h pump out), and a 21 h stabilization phase. The assumed WASHR performance was based on the results of the bench-scale tests at the assumed removal rates of 80% COD and 92% TSS during the contact stage and 58% COD and 47% TS during the stabilization stage. The capital costs included allowances for a single contact phase tank (200 m^3^) and a four-celled stabilization tank (400 m^3^ total volume), the necessary aeration system and blowers, pumps and piping, and other appurtenances. The upgrades required for the WWTP’s bioreactors, oxygenation system, anaerobic digesters, and biogas handling systems were considered for all three WWW treatment options. The projected upgrades were developed based on the typical design guideline requirements for Ontario’s municipal WWTPs [1]. Table 5 presents a summary of the capital, O&M, LCCs, and GHG emissions for the three upgrade options, for both the short- and long-term scenarios.

The LCC assessment confirms the economic viability of the WASHR, with the overall costs for both the short-term (CAD 47.88/m^3^) and long-term (CAD 67.31/m^3^) scenarios being significantly less than co-treatment in the liquid treatment train (CAD 54.15/m^3^ and CAD 89.04/m^3^ for the short- and long-term scenarios) and slightly higher than anaerobic co-treatment (CAD 43.37/m^3^ and CAD 54.08/m^3^). Furthermore, from a greenhouse gas (GHG) emissions perspective, the WASHR process would result in 959–960 tons of eCO_2_/yr, which is significantly less than anaerobic co-treatment (2204–2205 tons of eCO_2_/yr). Therefore, the WASHR process provides a good balance between cost effectiveness and minimizing the environmental impact.

## 5. Conclusions

A novel pre-treatment process (WASHR) is developed to optimize the co-treatment of hauled, high-strength wastewaters. The process is a combination and modification of the contact stabilization and SBR treatment processes and is operated in batch mode to accommodate the batch nature of WAS wasting and the receipt of hauled loads to municipal WWTPs. The WASHR process can be integrated into most municipal WWTPs, provided there is a suspended growth, aerobic treatment process that generates a WAS stream. The advantages of the WASHR process include that, although it is a biological treatment system, it can be brought online as needed, with no start-up or acclimation period needed. It also utilizes waste streams from the WWTP (WAS as well as digester supernatant, if available or needed), reducing costs. However, the capacity of the WASHR process is limited by the available WAS wasting rate from the WWTP’s main liquid treatment train.

Bench-scale trials confirm that the WASHR process, compared to direct co-treatment, can reduce the COD and TSS loadings into the WWTP’s liquid treatment train by more than 81% and 92%, respectively, and into the solid’s treatment train by more than 59% and 30%, respectively. Furthermore, the implementation of the WASHR pre-treatment system could reduce or eliminate the operational concerns associated with direct co-treatment, including the development of septic conditions and odor generation in the primary clarifiers, the low operating DO in the bioreactors, the excessive sludge yield reducing operating SRT, digester overloading and souring, and exceeding the capacity of the biogas handling system, which results in safety concerns.

A detailed economic and environmental impact analysis, using a case study approach, confirmed that the pre-treatment WASHR process has a lower cost than that in the direct co-treatment in the liquid treatment train and a smaller GHG footprint than that in the direct co-digestion in the solids treatment train. When incorporating the potential for carbon offsetting, the WASHR process has the potential to significantly reduce both GHG emissions and LCC costs compared to direct co-treatment.

## 6. Patents

A PCT patent (PCT/CA2022/050507 (WO 2022/204823)) was filed in April 2022 as a result of this study. This manuscript covers the novel method of high-strength wastewater treatment in detail.

## Figures and Tables

**Table 1:** Characteristics of the WWW and anaerobic digesters supernatant used in the bench-scale WASHR treatment trials.

Parameter	WWW	Digester Supernatant
COD (mg/L)	163,000	1200
TOC (mg/L)	57,300	259
TSS (mg/L)	71,600	556
VSS (mg/L)	51,600	438
TAN (mg/L)	10.9	246
TP (mg/L)	30.2	32.5

**Table 2:** Operating conditions for WASHR system trial runs.

Parameter	Unit	Run A	Run B	Run C	Run D
Contact Stage					
MLSS_o_	mg/L	1488	1488	1488	1488
MLVSS_o_	mg/L	1244	1244	1244	1244
Operating Volume	L	14	14	14	14
Reaction Cycle Duration	h	3 and 6	3 and 6	3 and 6	3 and 6
Settle Cycle Duration	h	0.5	0.5	0.5	0.5
Stabilization Stage					
Operating Volume	L	6	6	6	6
Aeration Cycle Duration	h	18	18	18	18
Feed Volumes					
WWW	L	0.14	0.28	0.42	0.56
Digester Supernatant	L	0.54	0.54	0.54	0.54
Initial Concentrations ^(1)^					
CODo	mg/L	1751	3381	5010	6639
TOC_o_	mg/L	583	1156	1729	2302
TSS_o_	mg/L	757	1473	2188	2904
VSS_o_	mg/L	551	1066	1582	2098
TAN_o_	mg/L	32.1	31.9	31.8	31.7
TKN_o_	mg/L	47.7	49.1	50.6	52.1
TP_o_	mg/L	2.07	2.37	2.66	2.96
Total Effluent Volumes ^(2)^					
Clarified Supernatant	L	7.1	7.1	7.1	7.1
Settled Biomass	L	6.9	6.9	6.9	6.9

Notes: MLSS: mixed liquor suspended solids; MLVSS: mixed liquor volatile suspended solids; TKN: total Kjeldahl nitrogen. ^(1)^ Initial overall concentrations in the reactor including contributions from WWW, digester supernatant, and liquid phase of the feed biomass (WAS from the WWTP). ^(2)^ Includes allowances for volumes removed for the purposes of sample analysis.

**Table 3:** Characteristics of the settled biomass pre- and post-bench-scale WASHR stabilization phase treatment of WWW.

Parameter	Unit	Run A	Run B	Run C	Run D
TS					
Pre-Stabilization	mg/L	4859	6177	7251	8230
Post-Stabilization	mg/L	4500	5480	6000	6860
Removal Rate	%	7.4	11.3	17.3	16.6
VS					
Pre-Stabilization	mg/L	3221	4143	4991	5594
Post-Stabilization	mg/L	3250	4020	4410	5080
Removal Rate	%	−0.9	3.0	11.6	9.2
TSS					
Pre-Stabilization	mg/L	4060	4685	5443	5826
Post-Stabilization	mg/L	4440	5297	5995	6390
Removal Rate	%	−9.3	−13.1	−10.1	−9.7
COD					
Pre-Stabilization	mg/L	5688	7439	9831	11,300
Post-Stabilization	mg/L	6920	7380	7980	9400
Removal Rate	%	−21.7	0.8	18.8	16.8

**Table 4:** Projected reductions in loadings to the WWTP’s treatment trains, achievable using the WASHR pre-treatment system compared with direct co-treatment of WWW.

Parameter	Reduction in Loadings to	
	Liquid Treatment Train	Solids Treatment Train
COD	81.4%	59.6%
TOC	83.6%	-
TSS	92.8%	30.2%
VS	-	47.9%
TAN	59.3%	-

**Table 5:** Comparison of the estimated capital costs, O&M costs, LCCs, and GHG emissions for the seasonal treatment of WWW at the case study facility.

Parameter	Unit	Liquid Treatment Train Co-Treatment	Solids Treatment Train Co-Treatment	WASHR Pre-Treatment
Short-Term				
Capital Cost	CAD	2,470,000	2,510,000	2,590,000
O&M Cost	CAD/yr	115,100	53,000	67,900
LCC Cost (15 Years)	CAD	3,898,800	3,122,800	3,447,500
Cost per Unit of Treated WWW	CAD/m^3^	54.15	43.37	47.88
GHG Emissions	eCO_2_ ton/yr	771	2204	959
Long-Term				
Capital Cost	CAD	8,520,000	5,580,000	6,770,000
O&M Cost	CAD/yr	118,200	55,400	69,500
LCC Cost (25 Years)	CAD	10,685,000	6,489,100	8,077,300
Cost per Unit of Treated WWW	CAD/m^3^	89.04	54.08	67.31
GHG Emissions	eCO_2_ ton/yr	771	2205	960

## Data Availability

Data are contained within the article.
